# A tubby-like protein CsTLP8 acts in the ABA signaling pathway and negatively regulates osmotic stresses tolerance during seed germination

**DOI:** 10.1186/s12870-021-03126-y

**Published:** 2021-07-17

**Authors:** Shuangtao Li, Zhirong Wang, Fei Wang, Hongmei Lv, Meng Cao, Na Zhang, Fengju Li, Hao Wang, Xingsheng Li, Xiaowei Yuan, Bing Zhao, Yang-Dong Guo

**Affiliations:** 1 Beijing Key Laboratory of Growth and Developmental Regulation for Protected Vegetable Crops, College of Horticulture, China Agricultural University, Beijing, 100193 China; 2 Beijing Academy of Forestry and Pomology Sciences, Beijing Academy of Agriculture and Forestry Sciences, Beijing, 100093 China; 3 Tianjin Academy of Agricultural Sciences, 300192 Tianjin, China; 4 Shandong Provincial Key Laboratory of Cucurbitaceae Vegetable Biological Breeding, Shandong Huasheng Agriculture Co. Ltd, Qingzhou, 262500 Shandong China

**Keywords:** *Cucumis sativus* L, E3 ubiquitin ligase, Salt stress, Osmotic stress, Transcription factor, Tubby-like protein

## Abstract

**Background:**

TLPs (Tubby-like proteins) are widespread in eukaryotes and highly conserved in plants and animals. TLP is involved in many biological processes, such as growth, development, biotic and abiotic stress responses, while the underlying molecular mechanism remains largely unknown. In this paper we characterized the biological function of cucumber (*Cucumis sativus* L.) Tubby-like protein 8 (CsTLP8) in *Arabidopsis*.

**Results:**

In cucumber, the expression of the tubby-like protein *CsTLP8* was induced by NaCl treatment, but reduced by PEG (Polyethylene Glycol) and ABA (Abscisic Acid) treatment. Subcellular localization and transcriptional activation activity analysis revealed that CsTLP8 possessed two characteristics of classical transcription factors: nuclear localization and trans-activation activity. Yeast two-hybrid assay revealed interactions of CsTLP8 with CsSKP1a and CsSKP1c, suggesting that CsTLP8 might function as a subunit of E3 ubiquitin ligase. The growth activity of yeast with ectopically expressed *CsTLP8* was lower than the control under NaCl and mannitol treatments. Under osmotic and salt stresses, overexpression of *CsTLP8* inhibited seed germination and the growth of *Arabidopsis* seedlings, increased the content of MDA (Malondialdehyde), and decreased the activities of SOD (Superoxide Dismutase), POD (Peroxidase) and CAT (Catalase) in *Arabidopsis* seedlings. Overexpression of *CsTLP8* also increased the sensitivity to ABA during seed germination and ABA-mediated stomatal closure.

**Conclusion:**

Under osmotic stress, CsTLP8 might inhibit seed germination and seedling growth by affecting antioxidant enzymes activities. CsTLP8 acts as a negative regulator in osmotic stress and its effects may be related to ABA.

**Supplementary Information:**

The online version contains supplementary material available at 10.1186/s12870-021-03126-y.

## Background

*Tubby* was first identified in the *tubby* strain of obese mice [1, 2]. TLPs are widespread in eukaryotes [3]. TLPs contain an about 270-amino acid tubby domain at the COOH-terminal, with a structure containing 12 anti-parallel β barrels and an intermediate hydrophobic α helix [4]. The NH_2_-terminal sequences of TLPs are quite divergent in animals, but conserved in plants, with most plant TLPs containing a conserved F-box domain in the NH_2_-terminal [3]. SKP1 (S-phase kinase-associated protein 1), CUL (Cullin), RBX1 (RING-box protein 1), and F-box protein can form the SCF complex [5, 6], an important component of E3 ubiquitin ligase. In *Arabidopsis* and wheat*,* AtTLPs and TaTULPs have been confirmed to interact with specific SKP1-like proteins [7–10], these findings indicate that plant TLPs might function as subunits of SCF complexes. In mammals, Tubby-like proteins constitute a unique family of transcriptional regulators [4], as the COOH-terminal region of Tubby binds double-stranded DNA, and the NH_2_-terminal regions of Tubby and TULP1 activate transcription. In plants, AtTLPs and CaTLP1 lack auto-activation activity [7, 11], but CaTLP1 binds to double-stranded DNA [11], suggesting that CaTLP1 may be a transcription factor.

Previous studies have assessed the efficacy of TLPs in response to biotic and abiotic stress in plants. In rice, all *OsTLPs* might involve in plant-pathogen interaction [12], besides, OsTLP2 can bind to the promoter of *OsWRKY13*, which encodes an activator important in rice resistance to bacterial infection [13], suggesting that OsTLP2 might function as a transcription factor to regulate biotic stress tolerance. In coffee and sugarcane *TLPs* were provided that play a role in response to biotic stress [14, 15].

In medicago, barley, maize, cassava, apple and chickpea, TLPs were reported to play a role in response to abiotic stress treatments or hormone treatment [11, 16–20]. Ectopically expressed *MdTLP7* increased growth activity of *E. coli* under NaCl, KCl, chilling, and heat treatment [21], and overexpression of full length or truncated *MdTLP7* (containing only the tubby domain) enhanced the stress tolerance of *Arabidopsis* to abiotic stresses [22], suggesting that *MdTLP7* functions in response to abiotic stress and the tubby domain of *MdTLP7* plays a key role in this response. The expression of *CaTLP1* was induced by dehydration, high salinity, and ABA [11]. Overexpression of *CaTLP1* increased the tolerance of tobacco and *Arabidopsis* to abiotic stress [11, 23]. Further study implied that CaTLP1 regulates the expression of ABA-mediated genes and stomatal closure by interacting with protein kinase [23]. AtTLP3 was released from the plasma membrane under mannitol, NaCl, or H_2_O_2_ treatment, suggesting that AtTLP3 functions in response to osmotic stress [7, 24].

In addition to response to biotic and abiotic stress, TLP also plays a role in plant growth and development. AtTLP9 functions during seed germination and participates in the ABA signaling pathway [9], and AtTLP3 redundantly functions with AtTLP9 in ABA- and osmotic stress-mediated seed germination [7]. The overexpression of *CaTLP1* improved shoot and root architecture, suggesting a key role for *CaTLP1* in plant development [11]. AtTLP2 regulates the biosynthesis of homogalacturonan [25], the major polysaccharide constituent of *Arabidopsis* seed coat mucilage [26], possibly through positive activation of UDP-glucose 4-epimerase 1 [25]. AtTLP11 interacts with AtNHL6, *AtTLP11* and *AtNHL6* exhibit antagonistic gene expression during seed germination [27]. AtNHL6 functions in abiotic stress-induced ABA signal transduction and biosynthesis, especially during early seedling development and seed germination [27, 28], and AtTLP11 may function by regulating AtNHL6. *ScTLP12* was identified as a putative gene responsible for leaf rolling in rye [29]. Zhang et al. explored the expression of *SlTLPs* in tomato fruit founded that *SlTLP1*, *SlTLP2*, *SlTLP4* and *SlTLP5* are ripening-related, and SlTLP1 and SlTLP2 may play a role in ethylene-dependent fruit ripening [30]. Our previous works imply that *SlTLFP8* regulated stomatal density through nuclear endoreduplication and affected water-deficient resistance and water-use efficiency [31].

Seed germination is a key process in the life cycle of higher plants, and play a function role in plant adapt to various environmental conditions, meanwhile, seed germination is stringently regulated by external and internal cues such as light, temperature, water, oxygen, plant hormone ABA, and GA [32–34]. Among these, the phytohormone abscisic acid (ABA) functions as a crucial signal to inhibit seed germination [35]. Drought and salt stresses are common limiting factors of seed germination. Drought and salt stress affects the seed germination process by decreasing water imbibition, including ROS (Reactive Oxygen Species) accumulation, altering enzymatic activities, and causing hormonal imbalances [36–38].

In this study, we found that *CsTLP8* transcriptionally responded to PEG, NaCl, and ABA treatments. Overexpression of *CsTLP8* inhibited the growth of yeast cells under NaCl and mannitol treatments. Overexpression of *CsTLP8* inhibited seed germination and the growth of *Arabidopsis* seedlings under osmotic and salt stresses, and increased ABA sensitivity in *Arabidopsis.* Under ABA, mannitol and NaCl treatment, overexpression of *CsTLP8* increased the content of MDA, and decreased the activities of SOD, POD and CAT in *Arabidopsis* seedlings. These results indicate that CsTLP8 acts as a negative regulator in salt stress and osmotic stress response by affecting antioxidant enzymes activities. Subcellular localization and trans-activation assays suggested CsTLP8 works as a transcription factor, and yeast two-hybrid assays revealed interaction of CsTLP8 with CsSKP1a and CsSKP1c, implying that CsTLP8 may also function as an E3 ubiquitin ligase.

## Results

### *CsTLP8* expression responds to abiotic stress

**Fig. 1:**
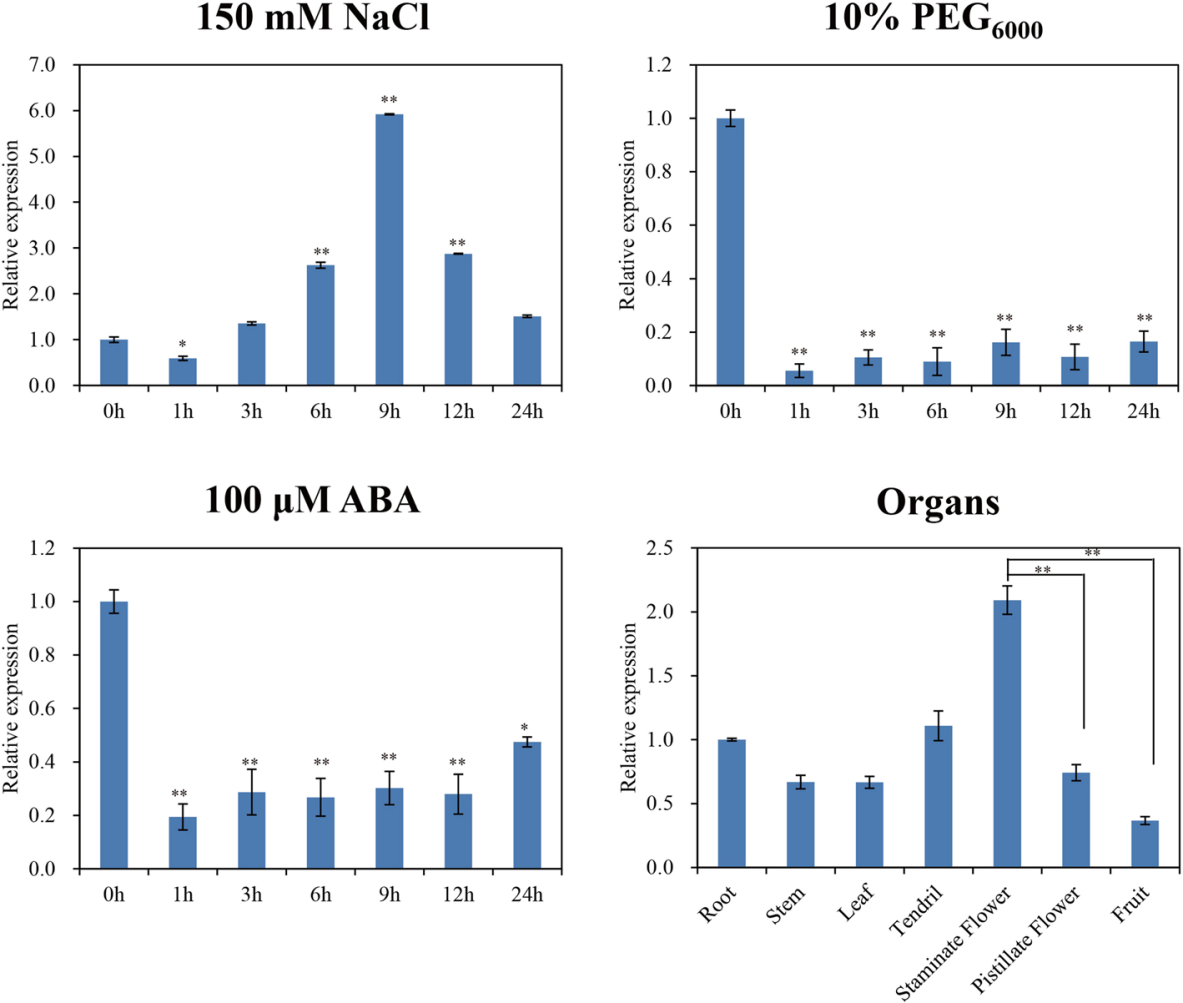
The expression pattern of *CsTLP8.* (**a**) The expression pattern of *CsTLP8* in the leaves under NaCl (150 mM) treatment. (**b**) The expression pattern of *CsTLP8* in the leaves under PEG_6000_ (10%) treatment. (**c**) The expression pattern of *CsTLP8* in the leaves under ABA (100 μM) treatments. (**d**) The expression pattern of *CsTLP8* in various organs (root, stem, leaf, staminate flower, pistillate flower, fruit and tendril). Values are means ± SD (n = 3), *P < 0.05 or **P < 0.01, Duncan’s test for multiple tests

To investigate the function of *CsTLP8* under abiotic stress in cucumber, we analyzed the expressions of *CsTLP8* under NaCl, PEG and ABA treatments. The expression level of *CsTLP8* was up-regulated about sixfold at 9 h after NaCl treatment (Fig. 1a), and reduced under both PEG treatment (Fig. 1b) and ABA treatment (Fig. 1c). Interestingly, the expression pattern of *CsTLP8* under ABA or PEG treatment was similar. We next explored the expression pattern of *CsTLP8* in seven organs (root, stem, leaf, staminate flower, pistillate flower and fruit). Results showed that *CsTLP8* was expressed in all the organs tested, the expression level of *CsTLP8* was highest in staminate flower and lowest in fruit, and there was no significant difference in the expression level of *CsTLP8* in vegetative organs, but significant differences in reproductive organs (Fig. 1d).

### Structural characteristics of CsTLP8

**Fig. 2:**
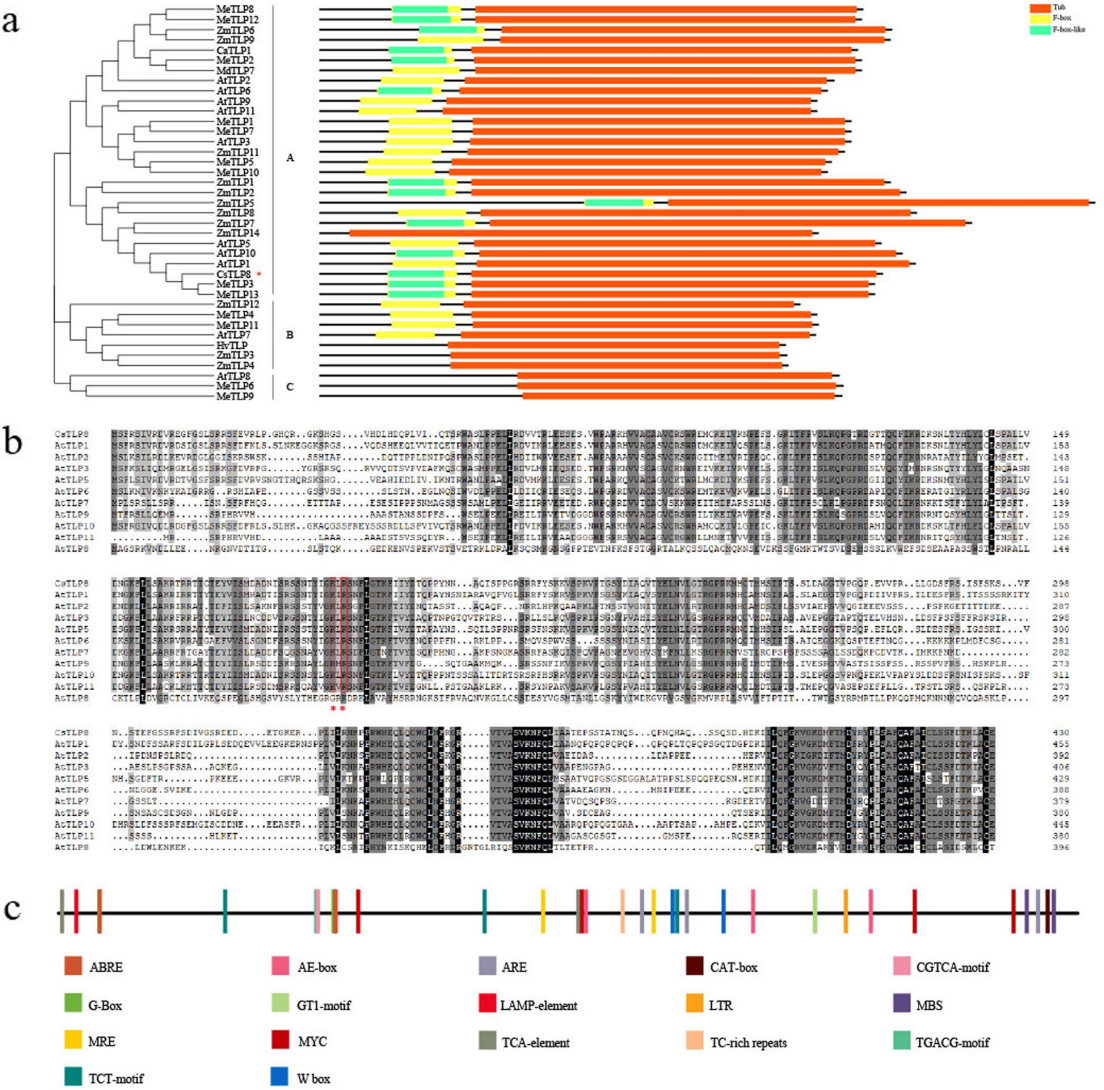
Structural characteristics of TLP. (**a**) Phylogenetic (left) and conserved domain (right) analyzes of CsTLP8, *Arabidopsis* AtTLPs, cassava MeTLPs, maize ZmTLPs, barely HvTLP, apple MdTLP7 and chickpea CaTLP1. (**b**) Alignment analyzes of CsTLP8 and Arabidopsis AtTLPs, red boxes and asterisk indicate the conserved PIP2 binding site. (**c**) Cis-elements analyzes of CsTLP8 promoter

To better understand the transcriptional regulation of *CsTLP8*, we extracted 2000 bp upstream sequences of *CsTLP8* and searched the PlantCARE database to identify the cis-elements. The *CsTLP8* promoter contains various cis-acting elements, including stress-responsive elements: TC-rich repeats (defense and stress response), MBS (drought response), GT-1 motif (salt response), LTR (temperature response), and ARE (anaerobic induction); hormone response elements: TCA-element (salicylic acid response), ABRE (Abscisic acid response), TGACG/CGTCA-motif (Methyl Jasmonate response); and light response elements: G-Box, MRE, AE-box, TCT-motif, and LAMP-element (Fig. 2c).

To better understand the function of CsTLP8, we analyzed its structural features. Conserved domain analysis showed that CsTLP8 harbor an F-box domain in the NH_2_-terminal region and a tubby domain in the COOH-terminal region (Fig. 2a). The 3D model of CsTLP8 revealed a highly conserved tubby domain, consisting of a closed 12-stranded β barrel and a central α helix (Fig. S1), the typical structure of the tubby domain. Phylogenetic analysis showed that CsTLP8 belongs to group A, and clustered in a clade with MeTLP3 and MeTLP13 (Fig. 2a). The presence of the F-box domain in the NH_2_-terminal region suggested that CsTLP8 may function as a subunit of the SCF complex. Multiple sequence alignment of CsTLP8 and AtTLPs revealed that CsTLP8 contains a conserved PIP_2_ (Phosphatidylinositol 4, 5-bisphosphate) binding site (Lys_188_/ Arg_190_) (Fig. 2b), which has previously shown to play a core role in the plasma membrane localization of Tubby [39] and AtTLP3 [24], indicating that CsTLP8 might locate in the plasma membrane.

### The subcellular localization and transactivation activity of CsTLP8

**Fig. 3:**
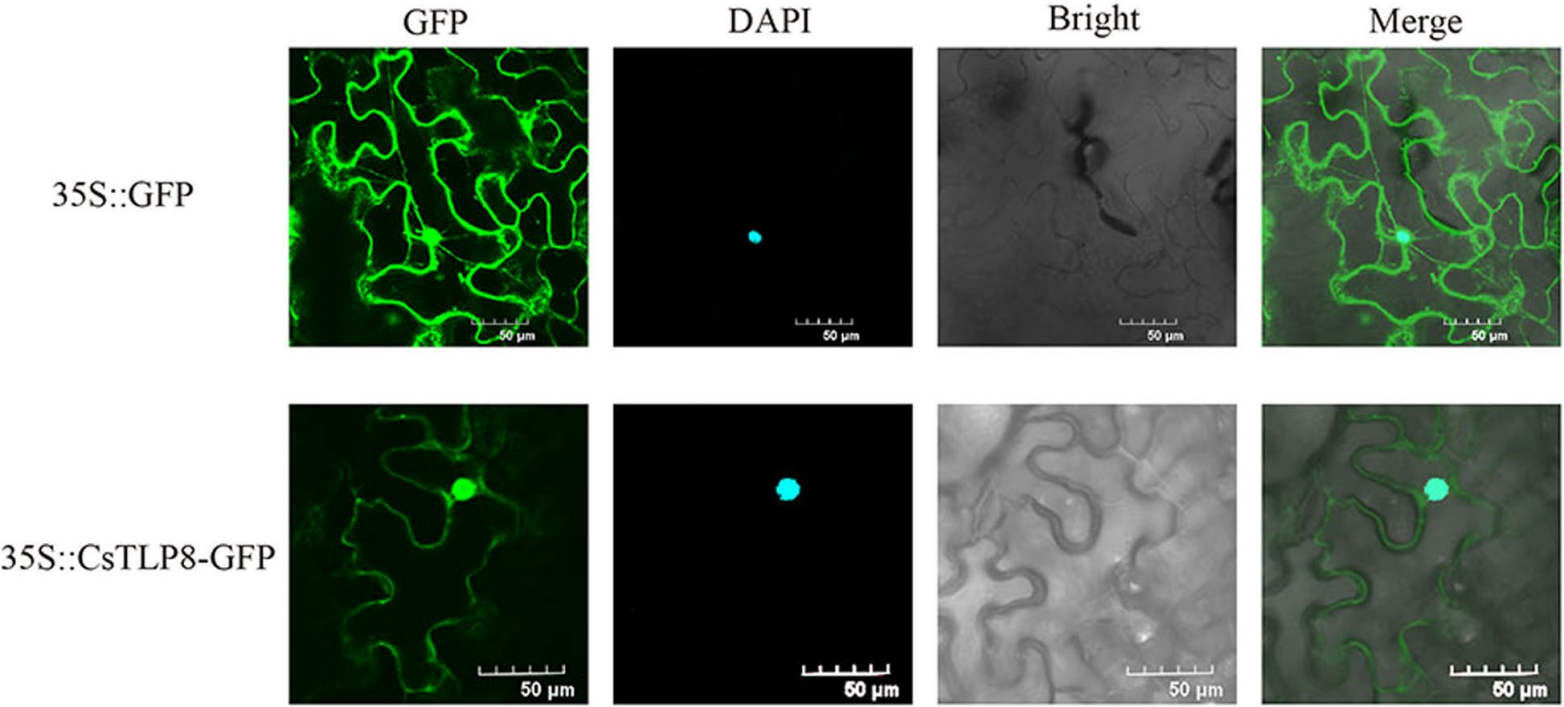
Subcellular localization of CsTLP8. GFP-CsTLP8 and GFP were transiently expressed in tobacco epidermal cells, respectively, and then observed with a confocal microscope

To further investigate the function of CsTLP8, we examined the subcellular localization of CsTLP8 in tobacco leaves. The fluorescence of CsTLP8-GFP fusion protein was observed in plasma membrane and nucleus, indicating that CsTLP8 localized in the plasma membrane and nucleus (Fig. 3), which is consistent with our conjecture.

**Fig. 4:**
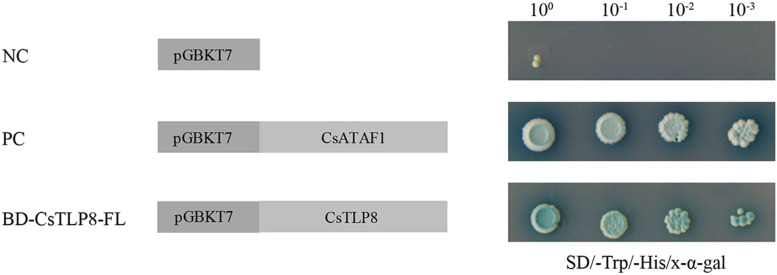
Transcriptional activity analyses of CsTLP8 by yeast one-hybrid. The yeast cells transformed with empty vector pGBKT7 (negative control, NC), pGBKT7-CsATAF1 (positive control, PC) or pGBKT7-CsTLP8 were streaked on SD/-Trp/-His/X-α-gal medium

To detect whether CsTLP8 had a transcription activation function, we analyzed the potential transcriptional activation activity of CsTLP8 by yeast one-hybrid assay. After incubated for 3 days, yeast cells transformed with pGBKT7-*CsTLP8* or pGBKT7-*CsATAF1* (positive control) survived well in SD/-Trp/-His/X-α-gal medium and turned blue, indicating activation of the reporter genes (Fig. 4). This result showed that CsTLP8 has auto-activation activity, and combined with the nuclear localization of CsTLP8, it is likely that CsTLP8 can function as a transcription factor in cucumber.

### The growth performance of yeast strains carrying CsTLP8 decreases under osmotic and salt stresses

**Fig. 5:**
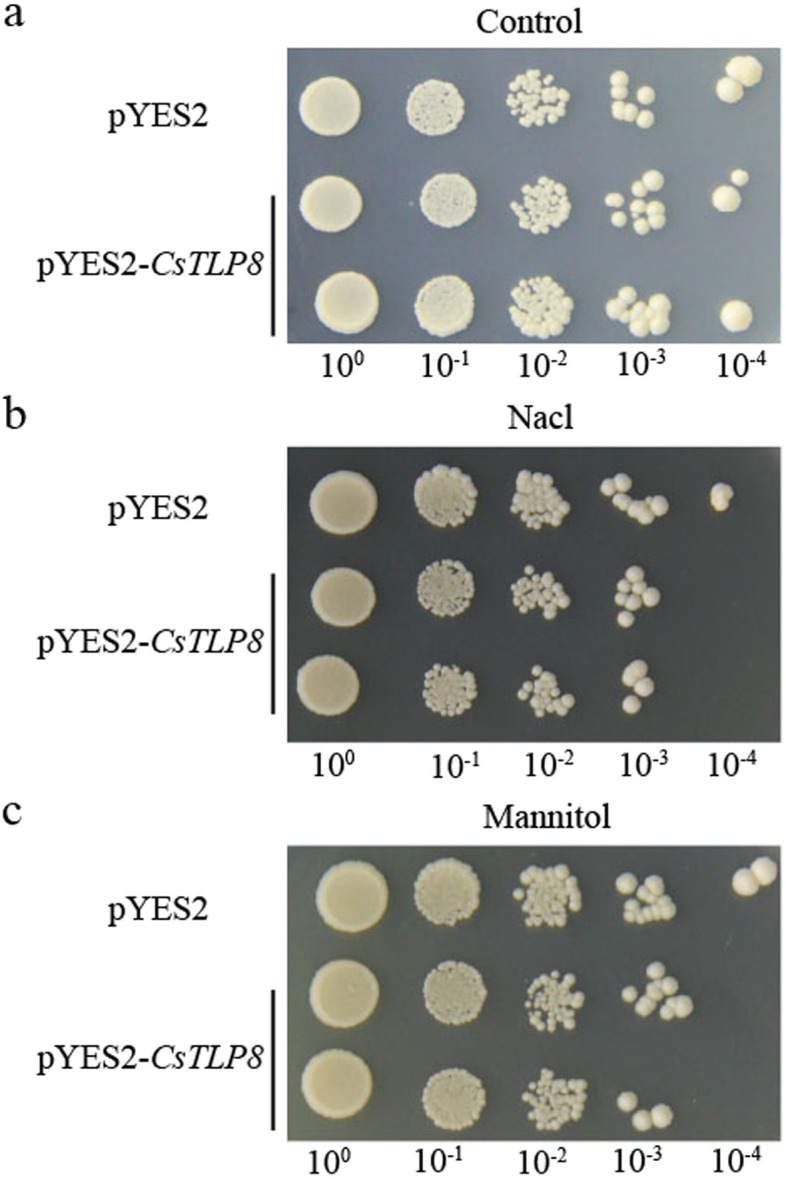
Transformed yeast strains had reduced growth activity under salt and drought stresses. The growth activity of yeast cells was tested by yeast spot assay when grown under control (**a**), 250 mM NaCl (**b**) and 300 mM mannitol (**c**) conditions on solid media

To assess the role of CsTLP8 under osmotic and salt stresses, *CsTLP8*-overexpressing yeast cells were generated, and the growth performance of these transgenic yeast cells was examined under osmotic and salt stresses by spot assays. Under normal conditions, there was no significant difference in growth performance for yeast cells transformed with pYES2-*CsTLP8* or empty vector pYES2 (control) (Fig. 5a). However, the growth of *CsTLP8*-overexpressing yeast cells was weaker than the control on medium supplemented with 250 mM NaCl or 300 mM mannitol (Fig. 5b-c), suggesting that overexpressing of *CsTLP8* significantly reduced the abiotic stress tolerance of yeast cells.

### Overexpression of *CsTLP8* decreases the tolerance to osmotic and salt stresses during seed germination in *Arabidopsis*

**Fig. 6:**
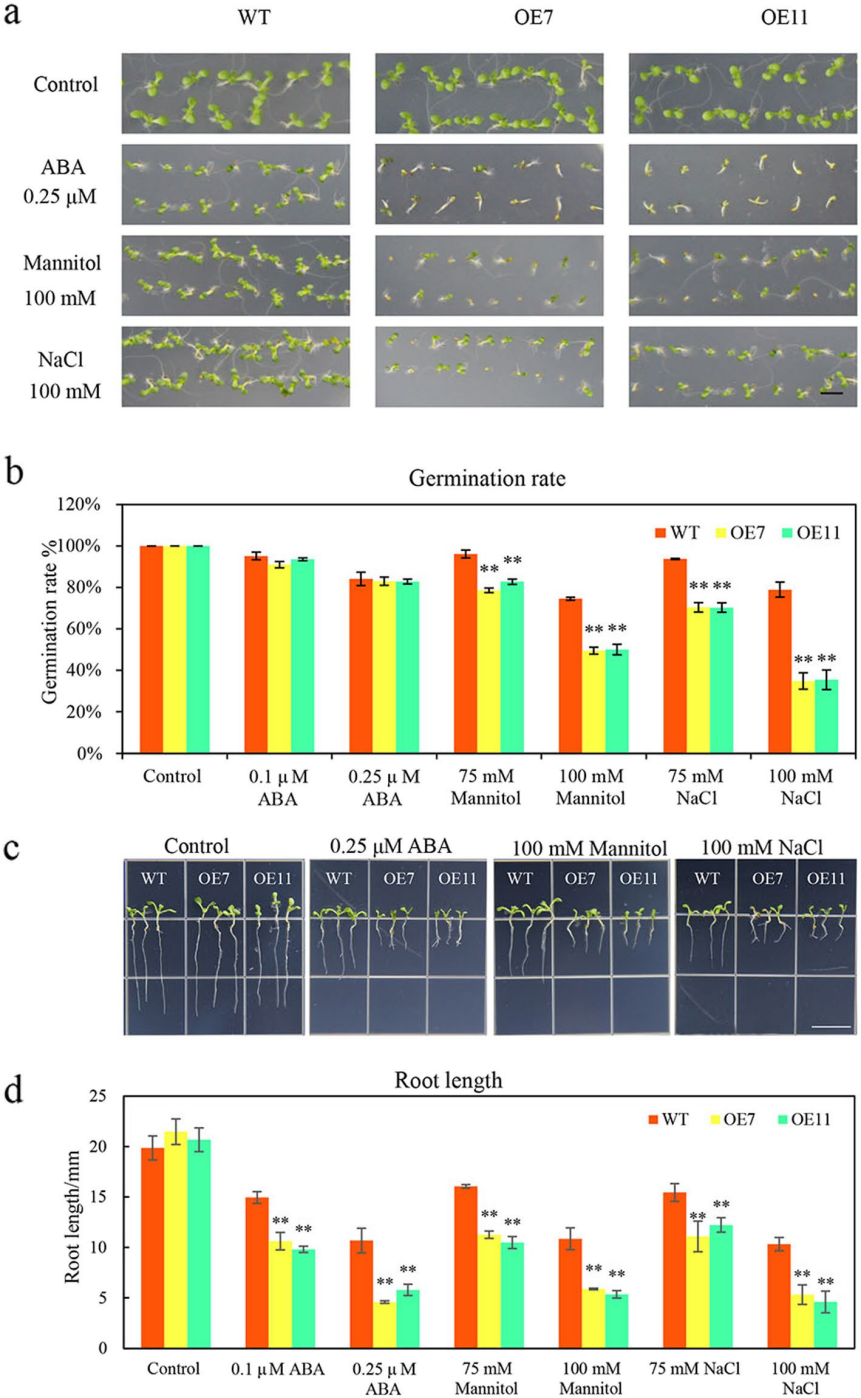
Overexpression of *CsTLP8* increased the sensitivity to ABA and decreased the tolerance to osmotic and salt stresses during seed germination in *Arabidopsis*. (**a**) Seeds of Col-0, OE7 and OE11 were surface sterilized, stratified for 3 d, and germinated on 1/2 MS media supplemented without or with ABA, mannitol or NaCl. Bar = 1 cm. (**b**) Quantification of corresponding germination rates. (**c**) Seeds of Col-0, OE7 and OE11 were sown for 4 days on half-strength MS medium and then transferred to half-strength MS medium with different concentrations of ABA, NaCl, or mannitol for 7 days. Bar = 1 cm. (**d**) Quantification of corresponding seedling root length. Values are means ± SD (*n* = 3), *P < 0.05 or **P < 0.01, Duncan’s test for multiple tests

To further investigate the biological function of *CsTLP8,* we ectopically expressed the coding sequences of *CsTLP8* in wild type *Arabidopsis* (Col-0). Two transgenic lines, OE7 and OE11, were selected for further study (Fig. S2). As shown above, the expression of *CsTLP8* responded to PEG and NaCl treatments, and ectopic expression of *CsTLP8* reduced growth performance of yeast cells under osmotic and salt stresses. Therefore, we measured the seed germination and seedling root length of wild type plants and transgenic lines on MS medium supplemented with different concentrations of mannitol and NaCl. Under normal conditions, the seedling growth and seed germination rate of transgenic lines were similar to those of wild type (Fig. 6). Under mannitol and NaCl treatment, the inhibition of seed germination and seedling growth of transgenic lines were more significant than those of wild lines. On medium containing 75 mM mannitol, the germination rates of wild type and two transgenic lines were 96%, 78%, and 82%, respectively (Fig. S3 & Fig. 6b), and the length of root were 16.0 mm, 11.3 mm and 10.5 mm, respectively (Fig. S3 & Fig. 6c-d). With the increase of mannitol concentration, the inhibition of seed germination increased. On the medium containing 100 mM mannitol, the germination rates of wild type and two transgenic lines were 74%, 49% and 50%, respectively, (Fig. 6a-b), and the root lengths were 10.7 mm, 5.9 mm, and 5.3 mm, respectively (Fig. 6c-d). At 75 mM NaCl, the germination rates of wild type and transgenic lines were 93%, 70.4% and 70.2%, respectively, (Fig. S3 & Fig. 6b) and root lengths of wild type and transgenic lines were 15.5 mm, 11.0 mm, and 12.2 mm, respectively (Fig. S3 & Fig. 6c-d). At 100 mM NaCl, 34%-38% seeds of transgenic lines germinated, while 78% seeds of wild type germinated (Fig. 6a-b) and the root lengths of wild type and transgenic lines were 10.3 mm, 5.3 mm and 4.6 mm, respectively (Fig. 6c-d). All these results indicated that CsTLP8 functions during seed germination and seedling growth in *Arabidopsis*.

### Overexpression of* CsTLP8* increases ABA sensitivity in *Arabidopsis*

**Fig. 7:**
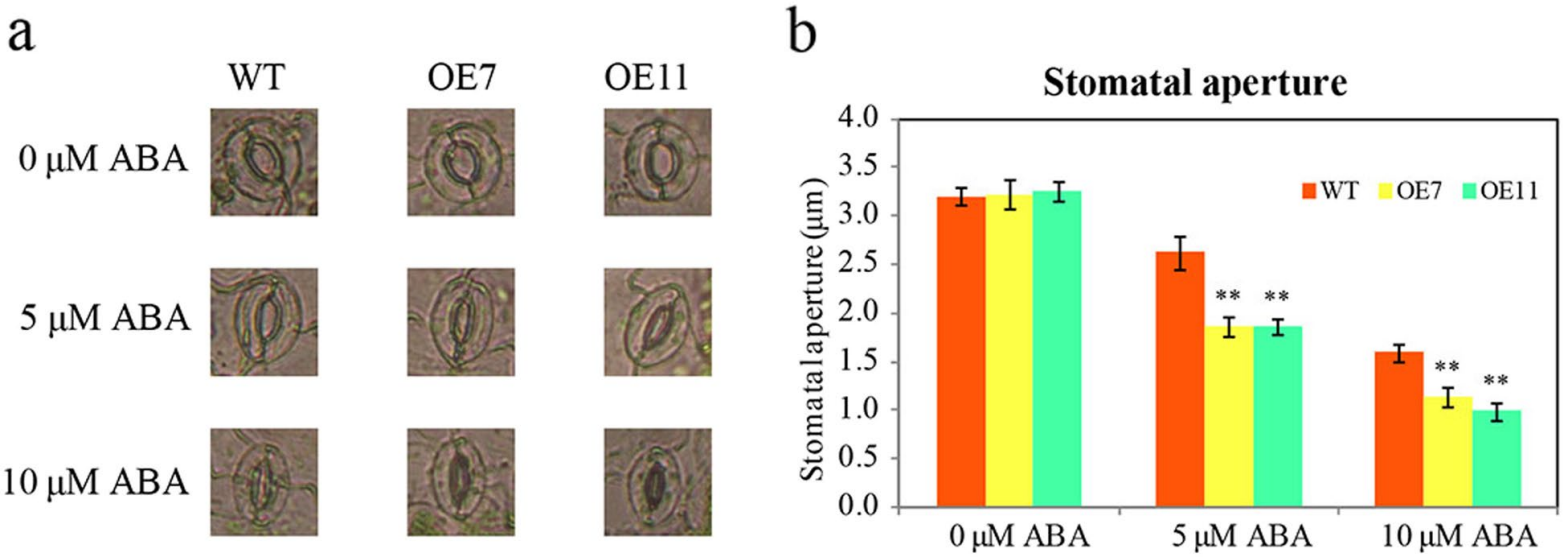
Overexpression of *CsTLP8* increased the sensitivity to ABA in *Arabidopsis.* Stomatal apertures in wild type and transgenic plants after ABA treatment. Representative images were taken under a microscope (**a**) and the stomatal apertures were measured (**b**). Values are means ± SD (n = 3), *P < 0.05 or **P < 0.01, Duncan’s test for multiple tests

As the expression of *CsTLP8* responded to ABA treatments, we next measured the response to ABA in transgenic lines. We employed seed germination rate, seedling root length and stomata aperture as indicators of ABA sensitivity. The seeds of wild type and transgenic lines germinated on MS medium supplemented with different concentrations of ABA. Under normal conditions, the seed germination rate and seedling growth of transgenic lines were similar to those of wild type. With the increase of ABA concentration, the seed germination rate decreased and the inhibition on seedling growth increased. There was a significant difference in seedling growth between transgenic lines and wild type, although there was no significant difference in seed germination rate between *CsTLP8* overexpression plants and wild type (Fig. 6a-b and Fig. S3). At 0.1 μM ABA, the root length of wild type was 1.4–1.5 times that of transgenic lines (Fig. S3 & Fig. 6c-d). The seedling growth difference was more significant at 0.25 μM ABA, and the root length of wild type was 1.8–2.3 times that of transgenic lines (Fig. 6c-d). To further examine the function of CsTLP8 to the ABA response, we measured stomatal apertures in transgenic plants, and wild-type plants in response to ABA. There was no difference in stomatal aperture of all the lines in the absence of ABA, but ABA treatment led to an increased stomatal closure in the transgenic lines, in the presence of 5 μM ABA, the stomatal aperture of wild type was 1.4–1.5 times that of transgenic lines; in the presence of 10 μM ABA, the stomatal aperture of wild type was 1.5–1.6 times that of transgenic lines (Fig. 7). These data suggesting that CsTLP8 positively regulates ABA-dependent stomatal movement. These results showed that overexpression of *CsTLP8* increased the sensitivity to ABA in *Arabidopsis.*

### Overexpression of* CsTLP8* reduce antioxidant enzymes activities in* Arabidopsis* seedling under osmotic and salt stresses

**Fig. 8:**
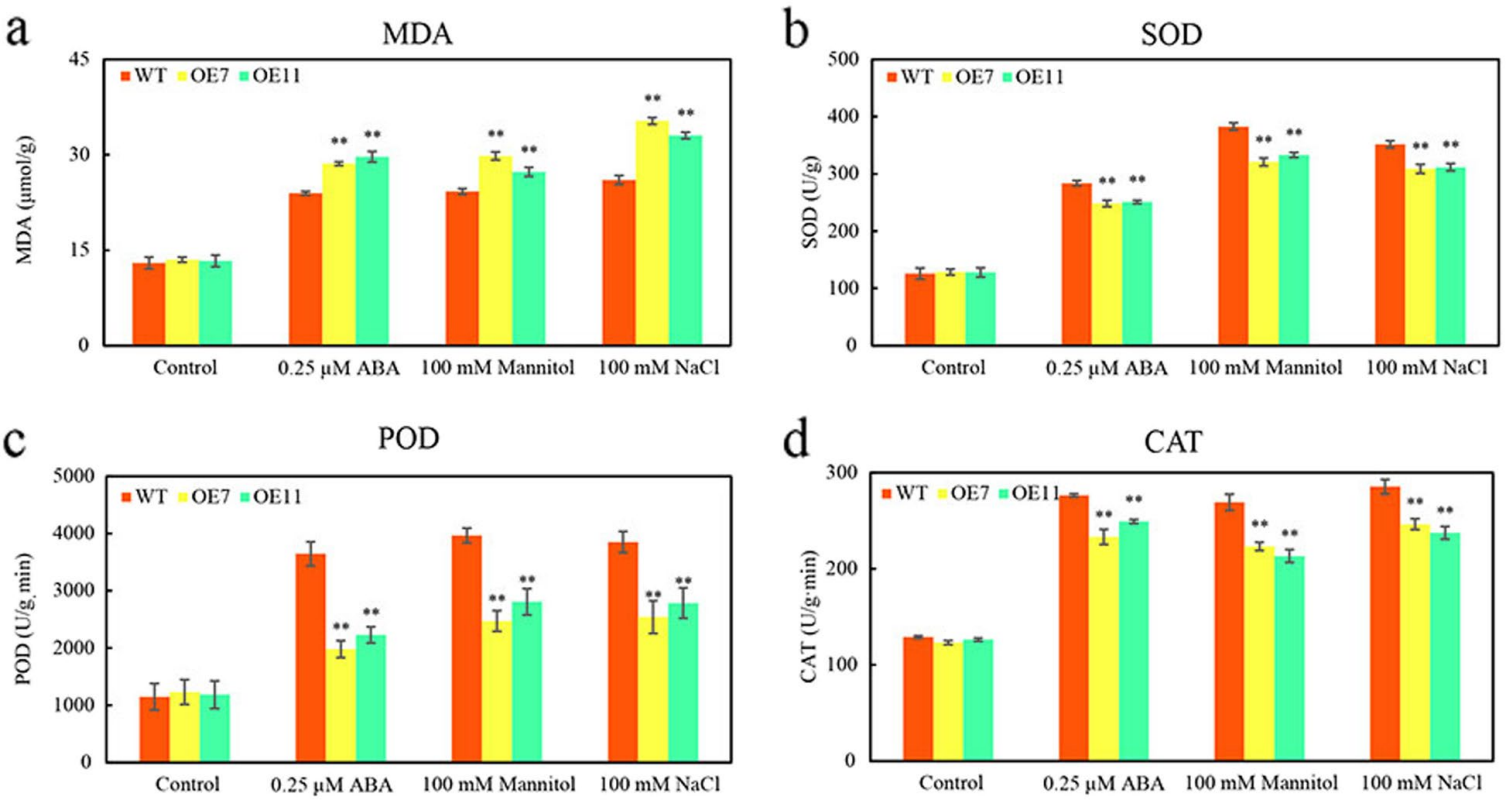
Overexpression of *CsTLP8* reduce antioxidant enzymes activities in *Arabidopsis* seedling under osmotic and salt stresses. (**a**) The content of MDA (Malondialdehyde) in wild type and transgenic lines under 0.25 μM ABA, 100 mM mannitol and 100 mM NaCl treatment. (**b**) The activity of SOD (Superoxide Dismutase) in wild type and transgenic lines under 0.25 μM ABA, 100 mM mannitol and 100 mM NaCl treatment. (**c**) The activity of POD (Peroxidase) in wild type and transgenic lines under 0.25 μM ABA, 100 mM mannitol and 100 mM NaCl treatment. (**d**) The activity of CAT (Catalase) in wild type and transgenic lines under 0.25 μM ABA, 100 mM mannitol and 100 mM NaCl treatment. Values are means ± SD (*n* = 3), *P < 0.05 or **P < 0.01, Duncan’s test for multiple tests

To evaluate physiological changes, the contents of MDA and activities of SOD, POD, CAT were measured following ABA, mannitol and salt treatment. Under normal conditions, there was no significant difference in MDA content between wild type and transgenic lines; under ABA, mannitol and salt treatment, the content of MDA in wild type and transgenic lines increased, and the content of MDA in wild type was significantly higher than that of transgenic lines (Fig. 8a). Under normal conditions, there was no significant difference in SOD, POD and CAT activities between wild type and transgenic lines; under ABA, mannitol and salt treatment, the activities of SOD, POD and CAT in all lines increased, and the activities of SOD, POD and CAT in transgenic lines were significantly higher than those of wild type (Fig. 8b-8c).

### CsTLP8 interacts with CsSKP1a and CsSKP1c

**Fig. 9:**
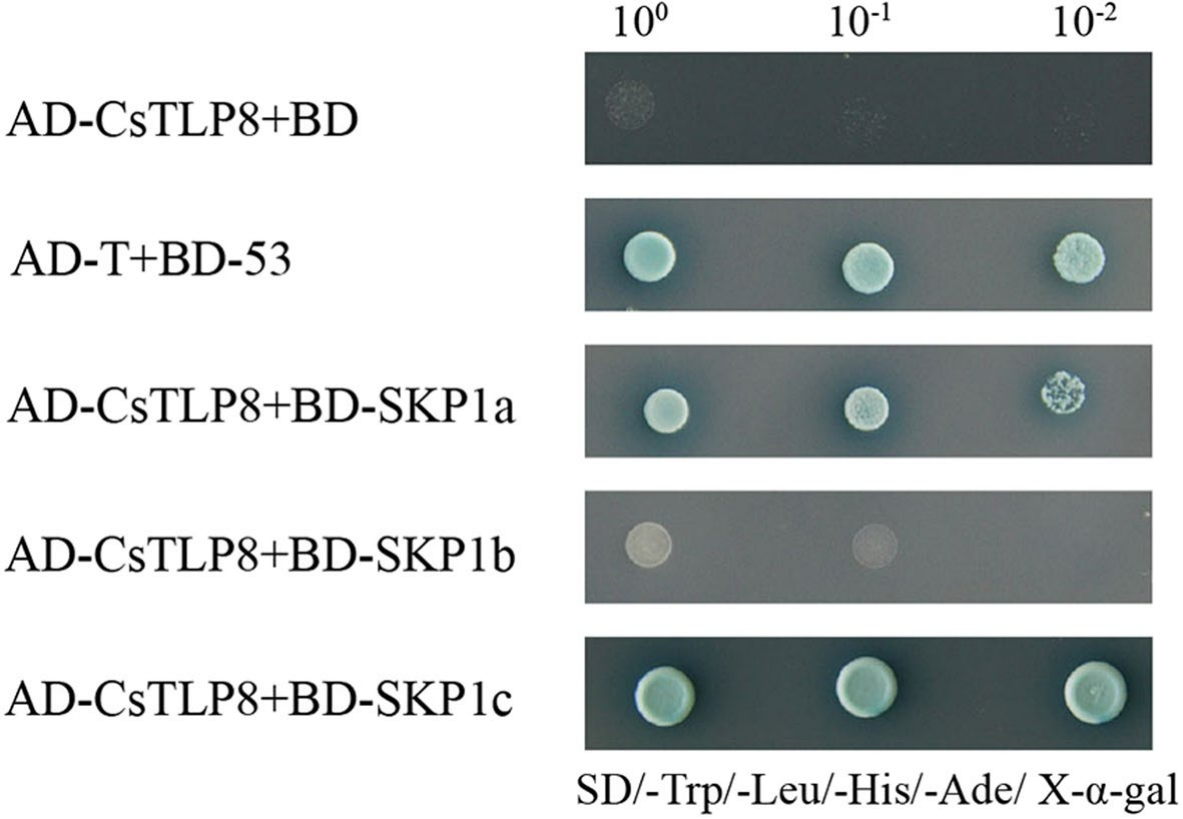
CsTLP8 interact with CsSKP1a and CsSKP1c. Protein–protein interaction analyzed by Yeast two-hybrid assay. The yeast cells transformed with BD + AD-CsTLP8 (negative control), BD-53 + AD-T (positive control) or BD-CsSKP1s + AD-CsTLP8 were streaked on SD/-Trp/-Leu/-Ade/-His/X-α-gal medium

We performed yeast two-hybrid assay to ask if CsTLP8 could interact with CsSKP1s as an F-box protein. The results suggest that CsTLP8 can interact with CsSKP1a and CsSKP1c (Fig. 9), indicating that CsTLP8 may function as an F-box protein, a subunit of SCF complex and participate in the degradation of the target protein.

## Discussion

*CsTLP8* was previously identified as a drought-related gene given its higher expression in drought-sensitive cultivar [40]. In our study, we found that expression of *CsTLP8* responded to salt and osmotic stresses (Fig. 1), overexpression of *CsTLP8* significantly reduced resistance of yeast cells to salt and osmotic stresses (Fig. 5), and seed germination and seedling growth were inhibited in *CsTLP8* overexpression plants under salt and osmotic stresses (Fig. 6 and Fig. S3). These findings implied that *CsTLP8* participates in the abiotic stress response during seed germination and seedling establishment. The process of germination occurs in three main phases regulated by hormones, reactive nitrogen species, and ROS [41]. ROS function as signaling molecules to regulate plant growth and development, but excess ROS could damage the structures of DNA, protein, lipid, and other macromolecules in the seeds. Thus, ROS scavenging is pivotal for seed germination under stress conditions [42, 43]. ROS can induce lipid peroxidation, which results in the production of MDA. In our study the content of MDA was used as a marker of oxidative stress, we found that overexpression of *CsTLP8* increased the level of oxidative stress in *Arabidopsis* seedling under osmotic and salt stresses. On the other hand, overexpression of *CsTLP8* reduced antioxidant enzymes activities in *Arabidopsis* seedling under osmotic and salt stresses. These results indicated that *CsTLP8* might regulate seed germination under abiotic stress by affecting antioxidant enzymes activities. As a crucial phytohormone, ABA plays a key role in many aspects of plants, including seed dormancy and germination, root growth, seedling development, and the adaptive response to environmental stresses. Overexpression of *CsTLP8* increased the sensitivity to ABA in *Arabidopsis,* such as shorter seedling root length and smaller stomatal aperture (Fig. 6, 7 and Fig. S3).

Cis-acting regulatory elements play key roles in the control of gene expression, and the *CsTLP8* promoter region contains various stress- and hormone-response elements (Fig. 2c). The observed different expression patterns under salt and PEG treatments (Fig. 1a-1b) suggest that the expression of *CsTLP8* can be regulated by different transcription factors via binding to different cis-acting elements under different stress signals. In *Arabidopsis*, *AtTLP3* and *AtTLP9* redundantly function during seed germination and early seedling development [7]. The phenotypes of *AtTLP3* knockout mutants and *AtTLP3* and *AtTLP9* double mutants under ABA and osmotic stress during seed germination are opposite to that of plants overexpressing *CsTLP8*, implying that *CsTLP8* plays a similar role during seed germination to *AtTLP3* and *AtTLP9*. Phylogenetic tree analysis clustered CsTLP8 in a clade with MeTLP13 and MeTLP3, and interestingly, the expression patterns of *CsTLP8* under NaCl treatment (Fig. 1a) is similar to that of *MeTLP3* as described by Dong et al. [19].

Structural features analysis and the 3D model revealed that CsTLP8 is a typical plant TLP (Fig. 2a), Multiple sequence alignment showed that CsTLP8 possesses a conserved PIP2 binding site (Fig. 2b), indicating that CsTLP8 may be located on the plasma membrane, which was confirmed by the results shown in Fig. 3. Tubby-PIP2 binding is reversible, as Tubby can interact with Gα_q_ a subset of G-proteins, resulting in the release of Tubby from the plasma membrane and enabling its translocation to the nucleus [39]. In *Arabidopsis,* AtTLP2 can interact with NF-YC3 (Nuclear Factor Y subunit C3) and be translocated into the nucleus [25]. AtTLP3 was released from plasma membrane under mannitol, NaCl, and H_2_O_2_ treatment [7], and the accumulation of CaTLP1 in nucleus was induced by dehydration stress [11].

In our study, we confirmed that CsTLP8 has trans-activation activity (Fig. 4) and combined with the nuclear localization of CsTLP8 (Fig. 3), we speculate that CsTLP8 may act as a transcription factor in cucumber. Most plant TLPs contains an F-box domain, like CsTLP8. Previous studies showed that most AtTLPs (AtTLP1, 3, 6, 9, 10 and 11) and TaTLPs (TaTULP1, TaTULP3 and TaTULP4) can interact with specific ASKs (*Arabidopsis* Skp1-like proteins)/ TaSKPs [8, 10]. Consistent with those findings, our research has demonstrated that CsTLP8 can interact with CsSKP1a and CsSKP1c (Fig. 9), suggesting that CsTLP8 can act as a subunit of the SCF complex and play a role in post-transcriptional regulation of target proteins. All of these findings suggest that CsTLP8 not only functions in transcriptional regulation, but also plays a role in post-transcriptional regulation. This is not unique, as the MAP kinase ERK5 possesses a kinase domain in the NH_2_-terminal region and a transcriptional activation domain and a nuclear localization signal in the COOH-terminal region, allowing this protein to regulate transcription at the nucleus by either phosphorylation or interaction with transcription factors [44]. In mouse, UBE3A is known as an E3 ubiquitin ligase, which several targets have been identified, UBE3A also functions as a transcriptional regulator of the family of nuclear receptors by interaction with IRF (Interferon Regulatory Factor) [45].

In cotton, the expression of *GhERF38* was up-regulated by salt and drought treatment, and over-expression of *GhERF38* in *Arabidopsis* reduced plant tolerance to salt and drought stress[46]. The expression of *ABI4*, a pivotal transcription factor in the ABA signaling pathway, was induced under salt stress, and ABI4 negatively regulates salt tolerance in *Arabidopsis* [47]. The expressions of *GmLHY1a* and *GmLHY1b* were all induced by drought stress, GmLHY1a and GmLHY1b negatively control drought tolerance in soybean [48]. All above these imply that the stress response genes, the expression was up-regulated under stress conditions, might function as a negative regulator in response to stress. This is consistent with our results in this study, that the expression of *CsTLP8* was induced by NaCl treatment, but the overexpression lines exhibited an increased susceptibility to salt stress. *WRKY25*, *WRKY26,* and *WRKY33* mediate responses to heat stress by positively regulating the cooperation between the HSPs and MBF1c pathways [49], and the WRKY33-PIF4 regulatory loop also mediates H_2_O_2_ homeostasis, suggesting that WRKY33 responds to different stresses via different signaling pathways [50]. Similarly, CsTLP8 may function as a transcription factor or an F-box protein, which may allow CsTLP8 to participate in different signaling pathways to respond to different stresses.

## Conclusion

In our study, we found that *CsTLP8* functions in ABA- and osmotic stress-mediated seed germination. We further confirmed that CsTLP8 regulates osmotic stress-mediated seed germination by affecting antioxidant enzymes activities, and CsTLP8 participates in ABA signaling pathway. In addition, we identified that CsTLP8 could function as a transcription factor and a subunit of the SCF complex. Our work provides a new sight to study the molecular mechanism of CsTLP8 response to abiotic stress. Our work provides a new sight to study the molecular mechanism of CsTLP8 response to abiotic stress. And we will further explore the molecular mechanism of CsTLP regulating seed germination and responding to drought and salt stress.

## Materials and methods

### Sample preparation and total RNA extraction

Cucumber seedlings (*Cucumis sativus* L. Jinyan 4, from Tianjin Cucumber Research Institute, Tianjin, China. Permissions for all the materials used in this experience have been obtained) were grown in Yamasaki culture medium in a growth chamber (photoperiod: 16 h light/8 h dark; temperature: 24 °C light/18 °C dark; light intensity: about 125 μmol m^−2^ s^−1^; relative humidity: about 60%). To investigate the expression of *CsTLPs* under abiotic stress, 3-week-old cucumber seedlings were treated with either 150 mM NaCl, 10% PEG_6000_, or 100 μM ABA for 0 h, 1 h, 3 h, 6 h, 9 h, 12 h, and 24 h. The leaves from three individual plants were collected, and these samples were used to extract total RNA according to our previously published protocol [51].

### Quantitative real-time PCR

Quantitative real-time PCR (qPCR) was conducted as described by Zhao et al. [52]. Basically, qPCR was performed on a 7500 Real-time PCR System (Applied Biosystems), the 2^−△△CT^ quantification method was used, and *CsActin* was used as an inner control gene.

### Cloning and sequence analysis of *CsTLP8*

The full-length coding sequence of *CsTLP8* was amplified by PCR from cucumber cDNA using the primer pairs listed in Supplementary Table S1. Phylogenetic analyses were conducted with MEGA5. Multiple alignment analysis was performed with DNAMAN. Conserved domains were analyzed using the Pfam database (http://pfam.xfam.org/). TBtools was used to redraw the phylogenetic tree and conserved domains. The three-dimensional model of CsTLP8 was built by SWISS-MODEL (https://swissmodel.expasy.org). The putative cis- elements present in the promoter sequence were predicted using the online program PlantCARE (http://bioinformatics.psb.ugent.be/webtools/plantcare/html/search_CARE.html).

### Transactivation assay of CsTLP8

The coding sequence of CsTLP8 was inserted into the pGBKT7 vector (Addgene, Cambridge, MA, USA). The yeast strain Y2H Gold [53] was transformed with pGBKT7-*CsTLP8*, pGBKT7-*CsATAF1* (positive control) [54], or the empty vector pGBKT7 (negative control) as by Ma et al. [55]. The transformed yeast strains were spotted onto SD/-Trp/-His/X-α-gal plates.

### Subcellular localization of CsTLP8

The coding sequence of *CsTLP8* (without termination codon) was inserted between CaMV 35S and GFP into the pCAMBIA1302 vector (Addgene, Cambridge, MA, USA). The recombinant vector and the empty vector pCAMBIA1302 (control) were introduced into *Agrobacterium tumefaciens* GV3101 and then transferred to tobacco leaves. The abaxial epidermis of transgenic tobacco was analyzed by confocal microscopy (FV1000, Olympus) with bright field and fluorescence imaging. Cell nucleus was stained with DAPI (Sigma-Aldrich, D9542).

### Yeast two-hybrid assays

The coding sequences of *CsTLP8* and *CsSKP1s* were inserted into pGADT7 (Addgene, Cambridge, MA, USA) and pGBKT7 vectors, respectively, pGADT7-CsTLP8 + pGBKT7-CsSKP1s, pGADT7-T + pGBKT7-53 (positive control) and pGADT7-CsTLP8 + pGBKT7 (negative control) were co-transformed into yeast Y2H Gold cells as previously described [56]. The transformed yeast colonies were selected on DDO medium (SD/-Leu/-Trp). Single colonies of transformants growing on DDO medium were transferred to QDO medium (SD/-Trp/-Leu/-His/-Ade) supplemented with X-α-gal.

### Yeast ectopic expression assays

The coding sequence of *CsTLP8* was inserted into the pYES2 vector (Addgene, Cambridge, MA, USA). The yeast strain W303 [57] was transformed with the recombinant vector or the empty vector pYES2. Growth assays were performed as described by Ye et al. [58], and 10 μl yeast culture at different dilution ratios was dropped on YPDA (Yeast peptone dextrose adenine) medium supplemented with 250 mM NaCl or 300 mM mannitol. After 72 h of culture at 30℃, the growth situation of yeast cells was observed and recorded.

### Plasmid Construction and *Arabidopsis* transformation

The open reading frame of *CsTLP8* was inserted into the pBI121 vector (Addgene, Cambridge, MA, USA), and then the recombinant vector was integrated into Columbia wild type *Arabidopsis* (Col-0) by *Agrobacterium tumefaciens* (C58)-mediated transformation. The transgenic lines were identified by resistance to kanamycin antibiotic and PCR amplification (Fig. S2). Two transgenic lines, OE7 and OE11, were chosen for further study.

### Phenotype analysis

Seed germination was conducted as previously described [59]. Briefly, 50 sterilized seeds were sown on half-strength MS medium supplemented without or with different concentrations of ABA, NaCl, or mannitol. Seeds were stratified at 4 °C in dark for 3 days and then transferred to growth chamber (photoperiod: 16 h light/8 h dark; temperature: 22 °C light/18 °C dark; light intensity: 80 μmol m^−2^ s^−1^; relative humidity 60%). After 7 days germination, the seed germination rate was calculated. In addition, seeds were sown for 4 days on half-strength MS medium and then transferred to half-strength MS medium with different concentrations of ABA, NaCl, or mannitol for 7 days, then root length were determined.

### Measurement of MDA content and Antioxidant Enzymes Activities

*Arabidopsis* seeds were sown for 4 days on half-strength MS medium and then transferred to half-strength MS medium with different 0.25 μM ABA, 100 mM NaCl, or 100 mM mannitol, 7 days later, seedlings were collected for measurement of MDA content and antioxidant enzymes activities. The content of malondialdehyde (MDA) as indicator of lipid peroxidation was measured according to the method of Qi et al. [60], briefly, samples were homogenized in phosphate buffer solution, then the supernatant was incubated with 5% TBA (Thiobarbituric Acid) at 100℃ for 10 min. Absorbance was analyzed at 600, 532, and 450 nm. The SOD activity was measured by monitoring the reduction in absorbance of NBT (Nitro-blue Tetrazolium) at 560 nm, according to the method of Zhang et al. [61]. CAT activity was determined by monitoring the disappearance of H_2_O_2_ at 240 nm, according to the method of Zhang et al. [61]. POD activity was determined by monitoring the oxidation of guaiacol, according to the method of Zhang et al. [61].

### Stomatal aperture bioassay

Stomatal apertures were measured as previously described [54]. Briefly, fully expanded rosette leaves from 3-week-old plants were collected and incubated in buffer solution (50 μM CaCl_2_, 10 mM KCl, 10 mM MES (2—(N-morpholino) ethanesulfonic acid), pH 6.0) for 2.5 h in light. Then leaves were treated with ABA for 2 h. Subsequently, the abaxial epidermal were peeled off and observed under a light microscope (Olympus-IX71). The stomatal apertures were measured using Image J, 100 stomata were measured for each sample.

### Primer list

The primers used in this study are listed in Supplementary Table S1.

### Statistical analysis

Each experiment was repeated three times, and then the data were analyzed by Duncan’s multiple range test (P < 0.05) using SPSS 18.0 software (IBM Corp. Armonk, NY, USA).

## Supplementary Information

**Additional file 1:** **Figure S1** Three-dimensional model of CsTLP8.

**Additional file 2:** **Figure S2** The transgenic lines were identified by the screen of kanamycin antibiotics (a) and PCR amplification (b).

**Additional file 3:** **Figure S3** Seeds of Col-0, OE7 and OE11 germinated on 1/2 MS media supplemented without or with ABA, mannitol or NaCl, Bar=1 cm.

**Additional file 4: Table S1** Primers used in this research. 

**Additional file 5:** **Table S2** List of gene accession number.

## Data Availability

Sequence data for genes described in this study are available using the accession numbers listed in Supplementary Table S2. All data generated or analyzed during this study are included in this published article and its supplementary information files, and available from the corresponding author on reasonable request.

## Abbreviations

ABA: Abscisic Acid
ASK: *Arabidopsis* Skp1-like proteins
CAT: Catalase
CUL: Cullin
DAPI: 4',6-Diamidino-2-phenylindole
IRF: Interferon Regulatory Factor
MDA: Malonaldehyde
MES: 2—(N-morpholino) ethanesulfonic acid
NBT: Nitro-blue Tetrazolium
NF-YC3: Nuclear Factor Y subunit C3
PEG: Polyethylene Glycol
PIP2: Phosphatidylinositol 4, 5-bisphosphate
POD: Peroxidase
qPCR: Quantitative real-time PCR
RBX1: RING-box protein 1
ROS: Reactive Oxygen Species
SOD: Superoxide Dismutase
SKP1: S-phase kinase-associated protein 1
TBA: Thiobarbituric Acid
TLP/TULP: Tubby-like protein
