# Supplementary figures and images for: A tubby-like protein CsTLP8 acts in the ABA signaling pathway and negatively regulates osmotic stresses tolerance during seed germination
Source: BMC Plant Biol. 2021 Jul 17;21:340. doi: 10.1186/s12870-021-03126-y (PMC8286588; doi:10.1186/s12870-021-03126-y)

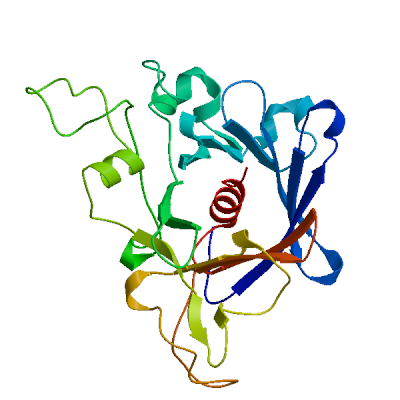

Supplement: Supplementary file 1 — Additional file 1: Figure S1 Three-dimensional model of CsTLP8. [file 12870_2021_3126_MOESM1_ESM.tif]

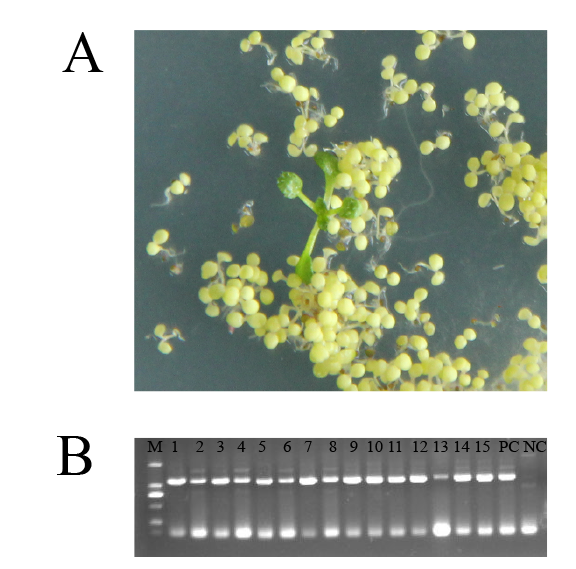

Supplement: Supplementary file 2 — Additional file 2: Figure S2 The transgenic lines were identified by the screen of kanamycin antibiotics (a) and PCR amplification (b). [file 12870_2021_3126_MOESM2_ESM.tif]

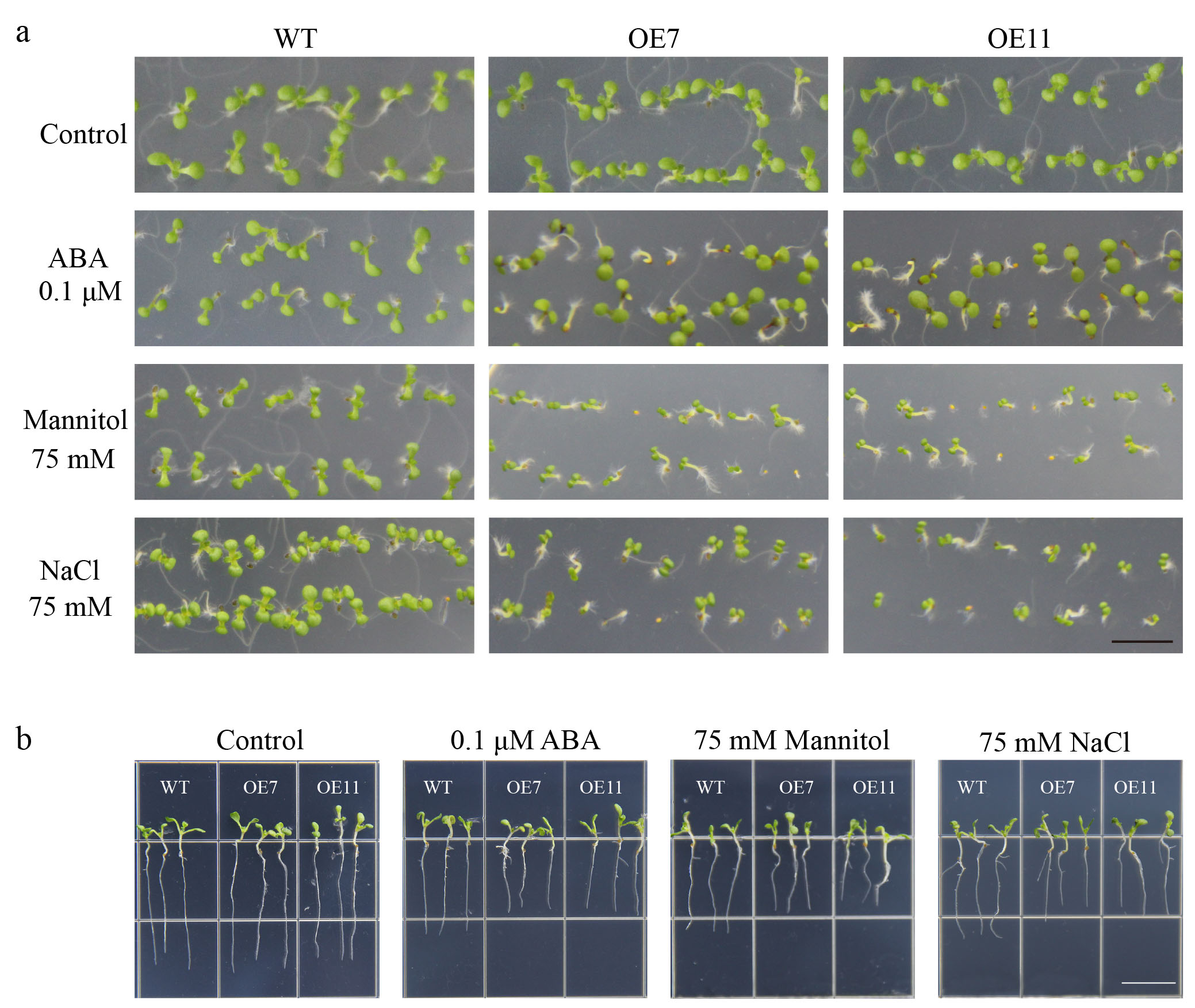

Supplement: Supplementary file 3 — Additional file 3: Figure S3 Seeds of Col-0, OE7 and OE11 germinated on 1/2 MS media supplemented without or with ABA, mannitol or NaCl, Bar=1 cm. [file 12870_2021_3126_MOESM3_ESM.jpg]
