# Supplementary material for: A tubby-like protein CsTLP8 acts in the ABA signaling pathway and negatively regulates osmotic stresses tolerance during seed germination
Source: BMC Plant Biol. 2021 Jul 17;21:340. doi: 10.1186/s12870-021-03126-y (PMC8286588; doi:10.1186/s12870-021-03126-y)
Supplement: Supplementary file 4 — Additional file 4: Table S1 Primers used in this research. [file 12870_2021_3126_MOESM4_ESM.docx]

Table **S1**. Primers used in this research

| Purpose | Gene | Primer |
| --- | --- | --- |
| qPCR | *CsTLP8* | \| F:ATGTCGTTTCGTAGTATAGTTCGTG \| \| --- \| |
|  |  | R:GCAGTCTCACCTCAAAACTACGC |
|  | *CsACTIN* | F:CCCCGATGGGCAGGTAATA |
|  |  | R:AAGAGCAGGACGAACAGCAGA |
| amplify | *CsTLP8* | F: ATGTCGTTTCGTAGTATAGTTCGTG |
|  |  | R：TTATTCACAAGCCAATTTGGTGTCA |
| OE construct | *CsTLP8* | F:GCTCTAGAGCATGTCGTTTCGTAGTATAGTTCGTG |
|  |  | R:CGGGATCCCGTTATTCACAAGCCAATTTGGTGTCA |
| PCR assay of OE plants | *CsTLP8* | F:CGCACAATCCCACTATCCTTC |
|  |  | R:ATCCAGACTGAATGCCCACAGG |
| Transactivation assay | *CsTLP8* | F:TCCCCCGGGATGTCGTTTCGTAGTATAGTTCGTG |
|  |  | R:CGCGGATCCTTATTCACAAGCCAATTTGGTGTCA |
| Subcellular localization | *CsTLP8* | F:ACCATGGTAGATCTGACTAGTATGTCGTTTCGTAGTATAGTTCGTG |
|  |  | R:AAGTTCTTCTCCTTTACTAGTTTCACAAGCCAATTTGGTGTCA |
| Yeast two-hybrid | *CsTLP8* | F:GTACCAGATTACGCTCATATGATGTCTTCTGGCCGGAAAATCACC |
|  |  | R:ACTGGCCTCCATGGCCATATGATTCAAATGCCCATTGGTTCTCCCT |
|  | *CsSKP1a* | F:TCAGAGGAGGACCTGCATATGATGTCTTCTGGCCGGAAAATCACC |
|  |  | R:TTCGGCCTCCATGGCCATATGATTCAAATGCCCATTGGTTCTCCCT |
|  | *CsSKP1b* | F:TCAGAGGAGGACCTGCATATGATGAGGATTGTTAACCTAAGATCAT |
|  |  | R:TTCGGCCTCCATGGCCATATGTTATTTACTGCCGCTAGTGC |
|  | *CsSKP1c* | F:TCAGAGGAGGACCTGCATATGATGTCGTCGTCTAAGAAGATCGTCC |
|  |  | R:TTCGGCCTCCATGGCCATATGCTACTCGAAGGCCCATTGATTCTCC |
| Yeast complementation assay | *CsTLP8* | F:CTTGGTACCGAGCTCGGATCCATGTCGTTTCGTAGTATAGTTCGTG |
|  |  | R:GCGGCCGTTACTAGTGGATCCTTATTCACAAGCCAATTTGGTGTCA |
