# Supplementary material for: A tubby-like protein CsTLP8 acts in the ABA signaling pathway and negatively regulates osmotic stresses tolerance during seed germination
Source: BMC Plant Biol. 2021 Jul 17;21:340. doi: 10.1186/s12870-021-03126-y (PMC8286588; doi:10.1186/s12870-021-03126-y)
Supplement: Supplementary file 5 — Additional file 5: Table S2 List of gene accession number. [file 12870_2021_3126_MOESM5_ESM.docx]

Table S2. List of gene accession number

| Gene name | Species | Database | Gene ID |
| --- | --- | --- | --- |
| *CsTLP8* | Cucumber | CuGenBD | Csa6G043990 |
| *CsSKP1a* | Cucumber | CuGenBD | Csa2G346050 |
| *CsSKP1b* | Cucumber | CuGenBD | Csa5G622630 |
| *CsSKP1c* | Cucumber | CuGenBD | Csa7G012950 |
| *AtTLP1* | Arabidopsis | TAIR | At1g76900 |
| *AtTLP2* | Arabidopsis | TAIR | At2g18280 |
| *AtTLP3* | Arabidopsis | TAIR | At2g47900 |
| *AtTLP5* | Arabidopsis | TAIR | At1g43640 |
| *AtTLP6* | Arabidopsis | TAIR | At1g47270 |
| *AtTLP7* | Arabidopsis | TAIR | At1g53320 |
| *AtTLP8* | Arabidopsis | TAIR | At1g16070 |
| *AtTLP9* | Arabidopsis | TAIR | At3g06380 |
| *AtTLP10* | Arabidopsis | TAIR | At1g25280 |
| *AtTLP11* | Arabidopsis | TAIR | At5g18680 |
| *MdTLP7* | Apple | NCBI | HM122708.1 |
| *CaTLP1* | Chickpea | NCBI | CAB88665.1 |
| *MtTLP* | *Medicago* | NCBI | XM-003588822.1 |
| *MeTLP1* | Cassava | Phytozome database | Manes.01G214100 |
| *MeTLP2* | Cassava | Phytozome database | Manes.02G095300 |
| *MeTLP3* | Cassava | Phytozome database | Manes.02G179400 |
| *MeTLP4* | Cassava | Phytozome database | Manes.03G100600 |
| *MeTLP5* | Cassava | Phytozome database | Manes.03G190900 |
| *MeTLP6* | Cassava | Phytozome database | Manes.04G100600 |
| *MeTLP7* | Cassava | Phytozome database | Manes.05G067900 |
| *MeTLP8* | Cassava | Phytozome database | Manes.05G157900 |
| *MeTLP9* | Cassava | Phytozome database | Manes.11G069900 |
| *MeTLP10* | Cassava | Phytozome database | Manes.15G017200 |
| *MeTLP11* | Cassava | Phytozome database | Manes.15G095300 |
| *MeTLP12* | Cassava | Phytozome database | Manes.18G023100 |
| *MeTLP13* | Cassava | Phytozome database | Manes.18G091900 |
| *ZmTLP1* | Maize | MaizeGDB | GRMZM2G001272 |
| *ZmTLP2* | Maize | MaizeGDB | GRMZM2G046816 |
| *ZmTLP3* | Maize | MaizeGDB | GRMZM2G062154 |
| *ZmTLP4* | Maize | MaizeGDB | GRMZM2G068586 |
| *ZmTLP5* | Maize | MaizeGDB | GRMZM2G108228 |
| *ZmTLP6* | Maize | MaizeGDB | GRMZM2G115701 |
| *ZmTLP7* | Maize | MaizeGDB | GRMZM2G129288 |
| *ZmTLP8* | Maize | MaizeGDB | GRMZM2G163726 |
| *ZmTLP9* | Maize | MaizeGDB | GRMZM2G176340 |
| *ZmTLP11* | Maize | MaizeGDB | GRMZM2G378907 |
| *ZmTLP12* | Maize | MaizeGDB | GRMZM2G435445 |
| *ZmTLP14* | Maize | MaizeGDB | GRMZM5G866954 |
| *HvTLP* | Barely | Ensembl Plants | HORVU3Hr1G061930 |
